# Acute Complex Type A Dissection associated with peripheral malperfusion syndrome treated with a staged approach guided by lactate levels

**DOI:** 10.1186/1749-8090-5-4

**Published:** 2010-01-28

**Authors:** Amna Suliman, Michael Dialynas, Hutan Ashrafian, Colin Bicknell, Maziar Mireskandari, Mohamad Hamady, Thanos Athanasiou

**Affiliations:** 1 Department of Cardiothoracic Surgery, Imperial College Healthcare NHS Trust, St Mary's Hospital, Praed Street, London W2 1NY, UK; 2 Regional Vascular Unit, Imperial College Healthcare NHS Trust, St Mary's Hospital, Praed Street, London W2 1NY, UK

## Abstract

Acute type A aortic dissection can be complicated by visceral malperfusion and is associated with a significant surgical morbidity and mortality. We describe a case of successful management of a complex acute type A dissection with mesenteric and lower limb ischemia treated with endovascular thoracic stenting and femoro-femoral crossover bypass grafting followed by aortic arch repair. To accomplish this, we applied a staged therapeutic approach using serial lactate measurements to assess the adequacy of peripheral perfusion and metabolic status prior to surgical repair of the proximal dissection.

## Background

Acute aortic dissection is amongst the most lethal surgical emergencies of the aorta. It results from a tear in the aortic wall intima that extends into the aortic wall media to create a false lumen and a dissection flap. Dissections of the ascending aorta are categorized as Type A according to the Stanford classification, and are complicated by visceral malperfusion in 16-33% of cases [1,2]. This is due to the antegrade propagation of the dissection from the ascending aorta to the level of the aortic visceral branches. These complex cases are associated with a significant mortality (up to 89% of cases), particularly in the presence of mesenteric ischemia (resulting in multi-organ failure) that renders surgical repair difficult [3,4]. Recent reports have suggested that physiological stabilization through the restoration of visceral perfusion by endovascular techniques as a beneficial strategy prior to dissection repair [5]. The extent of malperfusion however remains difficult to assess in view of the poor clinical signs which typically present at a late stage. The use of biomarkers such as serum lactate has therefore been suggested as potentially useful indicators of ischemia [6-8].

We describe a case of successful management of such a complex acute type A dissection with mesenteric and lower limb ischemia treated with endovascular thoracic stenting and femoro-femoral crossover bypass grafting followed by aortic arch repair. To achieve this, we applied a staged therapeutic approach using serial lactate measurements to assess the adequacy of peripheral perfusion and metabolic status prior to surgical repair of the proximal dissection.

## Case Presentation

A 63-year-old Japanese man presented with sudden onset chest pain radiating to his back and weakness in both lower limbs. Past medical history included mild coronary artery disease that did not require intervention, atrial fibrillation, secondary polycythemia associated with smoking, psoriasis and degenerative spondyloarthirits, and no history of other connective tissue disorders. There was no previous history of cerebrovascular or peripheral vascular disease. He was transferred to our institution over 12 hours from initial presentation, and was assessed by our multidisciplinary team (cardiothoracic surgeon, vascular surgeon and an interventional radiologist). On examination his blood pressure was 225/136 mmHg and there was clear ischemia of both lower limbs with bilateral absent femoral pulses. The sensory and motor function in the lower extremities was significantly reduced and abdominal examination was unremarkable.

**Figure 1:**
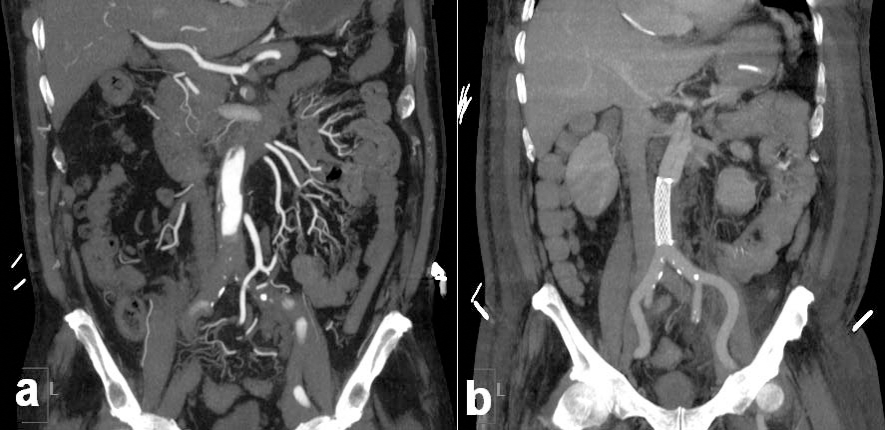
**(*a*) Pre-operative coronal view of the aorta and the aorto-iliac segment showing contrast in the aorta but no flow in the iliac arteries**. The dissection extended into both sides. (***b***) Post-operative coronal view of the same segment with uncovered stent in-situ demonstrating increased flow within the iliac system

**Figure 2:**
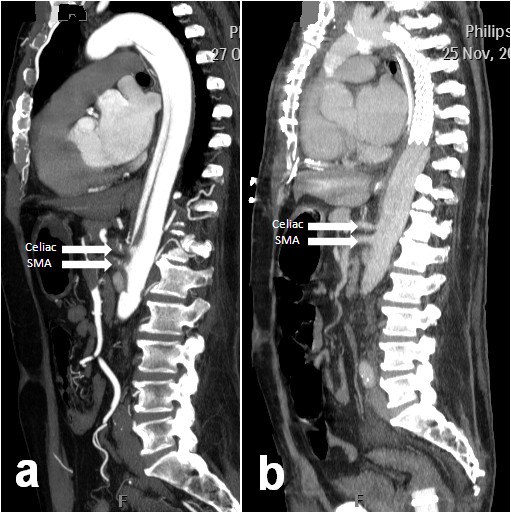
**(*a*) Pre-operative sagittal view of the thoracic aorta showing contrast within the true and false lumina**. Note near total occlusion of the celiac and superior mesenteric arteries (SMA) denoted by white arrows. (***b***) Post operative sagittal image of the same aortic segment with stent graft in-situ demonstrating increased flow within the celiac and superior mesenteric arteries (SMA) denoted by white arrows.

Computed Tomographic Angiography (CTA) revealed a complex type-A aortic dissection with the primary entry in the aortic arch leading to a dissection flap arising within the inferior aspect of the aortic arch and distal aorta extending to involve the entire thoracic aorta. The true lumen was small and severely narrowed beyond the level of the right renal artery, disappearing entirely just above the aortic bifurcation (Figure 1a and Figure 2a). No contrast could be visualized in the native iliac arteries and there was reduced blood flow in the celiac axis and the primary branches of the superior mesenteric artery which were perfused only by a very small channel of contrast seen extending from the true lumen. The transverse colon appeared thick-walled but both liver and spleen were normal. His left kidney was well perfused from the false lumen but there was no enhancement of the right kidney, which received its arterial supply from the true lumen. There was no involvement of the head and neck vessels or coronary arteries and there was no pleural or pericardial effusion.

Arterial bloods gas analysis revealed a mild acidosis (pH 7.34 with a base excess of -5.7) and an elevated lactate level of 11.9 mmol/lt. Blood pressure control was administered by beta-blockade and gylceryl-trinitrate infusion. Following stabilization, surgical management took place in 4 stages:

**1) Endovascular insertion of 2 stents: **Through a right axillary and bilateral common femoral approaches, a 150 mm covered stent graft (Medtronic, Santa Rosa, USA) was deployed into the thoracic aorta, distal to the left subclavian artery. A further covered stent (14 × 14 × 60 mm) (Medtronic, Santa Rosa, USA) was deployed in the infra-renal aorta, improving right but not left femoral circulation. The right axillary wound was temporarily closed with a conduit for cannulation use in the subsequent repair of the aorta. This was directly followed by femoro-femoral bypass grafting.

**2) Femoro-femoral bypass grafting: **An 8 mm Dacron graft was used for right to left femoro-femoral bypass restoring left lower limb perfusion. This resulted in a full complement of palpable pulses in both lower limbs.

**3) Stabilization in the Intensive Care Unit (ICU): **The patient was observed closely particularly with regards to any indicators of persisting mesenteric ischemia. The biomarker lactate played a key role in our management and was measured by taking regular peripheral arterial samples. Having previously been >10 mmol/lt, overnight the lactate fell to 7.2 mmol/lt, then 3.1 mmol/lt and by the next morning (during 8 hours period) returned to normal levels. The normalization of the lactate levels indicated the stabilisation of the patient's condition with resolution of the visceral and peripheral ischemia. Based on biomarker levels and clinical status, a decision was subsequently made to proceed to surgical repair of the dissection.

**4) Surgical repair of the aortic dissection: **Following median sternotomy and cannulation via the previous right-axillary artery conduit, cardiopulmonary bypass was instituted and the patient was cooled to 22°C. Antegrade cardioplegia and cerebral perfusion were applied. Total circulatory arrest time was 20 min and total bypass time was 120 min. The entry point tear was located, the hemi-arch was excised, the false lumen was obliterated with 6- interrupted Teflon felt pledgetted sutures. We specifically passed these pledgetted sutures through the proximal stent in the medial part of the descending thoracic aorta providing extra strength in these stitches and potentially reducing the risk of stent migration or creation of endoleak in this weak part of the aortic wall. A 28 mm Dacron conduit was then anastomosed (hemi-arch replacement) and the patient was rewarmed to 37°C. The chest was packed and left open for delayed closure, which was performed 48 h later.

The outcome of this staged approach was very successful (Figure 1b and Figure 2b) and our patient recovered well. His progress was complicated by a hospital-acquired pneumonia requiring prolonged intubation and formation of a tracheostomy. The total ITU stay was 33 days. He was gradually rehabilitated, and was discharged 40 days after admission.

## Conclusions

Approximately 25% of aortic dissections have evidence of peripheral malperfusion at presentation [2]. In cases of peripheral malperfusion syndrome, particularly involving the superior mesenteric artery, the operative mortality is significantly increased [9]. In these cases with such degree of metabolic disturbance, temporary postponement in surgical repair while peripheral reperfusion is re-established may prove beneficial [3,9].

**Figure 3:**
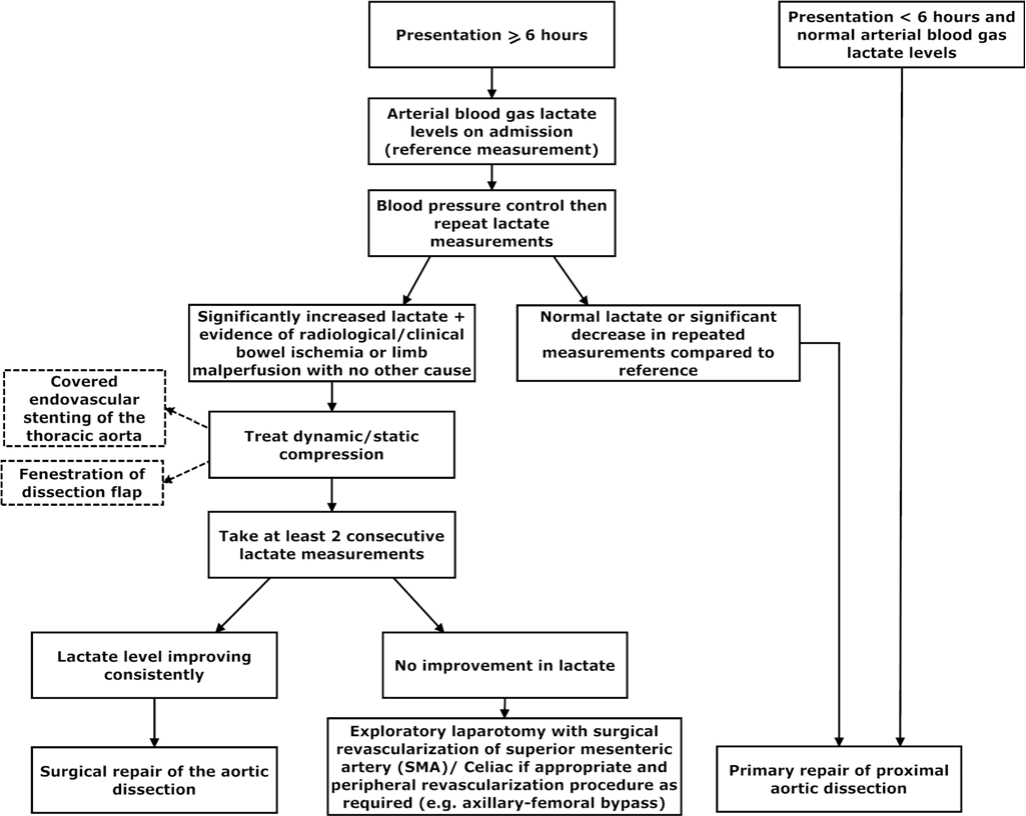
**Therapeutic approach in Type A dissection with peripheral malperfusion**.

Our patient did not have clinical signs of intestinal malperfusion (although there was significant peripheral ischemia). Lack of immediate symptoms in these patients can delay accurate diagnosis and management contributing to the high mortality. One possible treatment option includes initial endovascular fenestration of the infrarenal aorta [10]. In the last few years however, biomarkers (in particular serum lactate) have become a useful tool in assessing mesenteric ischemia. Our staged therapeutic approach (Figure 3) illustrates the diagnostic value of biomarkers in malperfusion, particularly where there is a delayed presentation.

If the initial lactate reading (measured quickly and simply from an arterial blood sample) is considerably high with no other cause and there is radiological or clinical evidence of bowel ischemia, revascularization using percutaneous endovascular techniques should first be carried out [5]. Following this, further serial lactate measurements should be taken to gauge the success of the intervention and monitor the improvement in peripheral malperfusion.

We recommend this method as D-lactate (a stereo-isomer of physiological L-lactate) is a sensitive marker for early mesenteric ischemia produced in large amounts by the overgrowth of gut microbial flora [6,8]. In view of the slow rate of enzymatic breakdown, it is a very sensitive early marker of the ischemic process (where the lactate levels may be subject to several factors including ischemia-related hepatic dysfunction) [6-8]. Our approach recommends that a consistent fall in lactate during this interim period may represent the ischemia as resolving. One can therefore perform a delayed repair of the proximal aortic dissection [11] providing a decreased intra-operative risk to the patient. Individuals with persistently high lactate levels may then require a revascularization procedure at that time rather than delaying intervention in anticipation of clinical signs. Differentiating between bowel ischemia and lower limb ischemia in the absence of clinical signs and a raised lactate can be based on radiological imaging.

A persistently raised lactate level associated with clinical or radiological evidence of bowel ischemia requires the treatment of the dynamic or static compression associated with the Type A dissection. This can be achieved by surgical revascularization, fenestration of the dissection flap or covered endovascular stenting of the thoracic aorta followed by closure of the dissection entry point with surgical repair (Figure 3).

If it is possible to attend a patient within 6 hours of a Type A dissection, then primary repair of the dissection is advised after locating the primary tear on preoperative CT scan (Figure 3). The optimal management for an acute type A dissection is entry closure and in cases of central aortic repair, distal organ ischemia can be managed through revascularization grafts such as axillary-femoral bypass. Endovascular stenting without entry closure for type A dissection has the risk of cardiac tamponade and in our case the entry point was closed during the arch repair. In more complex Type A dissections, a tailored multi-disciplinary strategy is required to address underlying risk in order to provide optimum perfusion and survival.

Our approach in using the biomarker lactate to guide our management of acute type A aortic dissection allows the restoration of an improved metabolic status before the insult of the total circulatory arrest (preserving the kidneys and bowel during the subsequent surgical dissection repair). It also has the potential to be extremely useful in terms of selecting patients who would be able to tolerate such complex operations and can improve patient outcomes in terms of morbidity and mortality.

## Consent

Written informed consent was obtained from the patient for publication of this case report and any accompanying images. A copy of the written consent is available for review by the Editor-in-Chief of this journal.

## Competing interests

The authors declare that they have no competing interests.

## Authors' contributions

AS participated in this case and contributed to its analysis. MD participated in this case and contributed to its analysis. HA participated in this case and contributed to its analysis. CB participated in this case and contributed to its analysis. MM participated in this case and contributed to its analysis. MH participated in this case and contributed to its analysis. TA participated in this case and contributed to its analysis. All authors read and approved the final manuscript.
